# Isolation and Characterization of Chi-like *Salmonella* Bacteriophages Infecting Two *Salmonella enterica* Serovars, Typhimurium and Enteritidis

**DOI:** 10.3390/pathogens11121480

**Published:** 2022-12-06

**Authors:** Addisu D. Teklemariam, Mona G. Alharbi, Rashad R. Al-Hindi, Ibrahim Alotibi, Abdullah A. Aljaddawi, Sheren A. Azhari, Ahmed Esmael

**Affiliations:** 1 Department of Biological Sciences, Faculty of Science, King Abdulaziz University, Jeddah 21589, Saudi Arabia; 2 Health Information Technology Department, Applied College, King Abdulaziz University, Jeddah 21589, Saudi Arabia; 3 Botany and Microbiology Department, Faculty of Science, Benha University, Benha 13518, Egypt; 4 Nebraska Center for Virology, University of Nebraska-Lincoln, Lincoln, NE 68583, USA

**Keywords:** bacteriophage, Chi-like phages, molecular characterization, *S*. Typhimurium, *S*. Enteritidis

## Abstract

*Salmonella enterica* Serovar Typhimurium and *Salmonella enterica* Serovar Enteritidis are well-known pathogens that cause foodborne diseases in humans. The emergence of antibiotic-resistant *Salmonella* serovars has caused serious public health problems worldwide. In this study, two lysogenic phages, STP11 and SEP13, were isolated from a wastewater treatment plant in Jeddah, KSA. Transmission electron microscopic images revealed that both phages are new members of the genus “*Chivirus*” within the family *Siphoviridae*. Both STP11 and SEP13 had a lysis time of 90 min with burst sizes of 176 and 170 PFU/cell, respectively. The two phages were thermostable (0 °C ≤ temperature < 70 °C) and pH tolerant at 3 ≤ pH < 11. STP11 showed lytic activity for approximately 42.8% (*n* = 6), while SEP13 showed against 35.7% (*n* = 5) of the tested bacterial strains. STP11 and STP13 have linear dsDNA genomes consisting of 58,890 bp and 58,893 bp nucleotide sequences with G + C contents of 57% and 56.5%, respectively. Bioinformatics analysis revealed that the genomes of phages STP11 and SEP13 contained 70 and 71 ORFs, respectively. No gene encoding tRNA was detected in their genome. Of the 70 putative ORFs of phage STP11, 27 (38.6%) were assigned to functional genes and 43 (61.4%) were annotated as hypothetical proteins. Similarly, 29 (40.8%) of the 71 putative ORFs of phage SEP13 were annotated as functional genes, whereas the remaining 42 (59.2%) were assigned as nonfunctional proteins. Phylogenetic analysis of the whole genome sequence demonstrated that the isolated phages are closely related to Chi-like *Salmonella* viruses.

## 1. Introduction

Yearly, *Salmonella* infection is responsible for 93.8 million cases and nearly 155,000 deaths of food poisoning globally, of which 85% of cases are associated with foodborne illness [1,2,3]. Approximately 2600 *Salmonella* serotypes are distinguished by the Kauffmann-White scheme [4,5]. Diverse groups of *Salmonella* species are found within this genus, including *Salmonella* bongori and *Salmonella* enterica; the latter is divided into six subspecies: *salamae*, *enterica*, *diarizonae*, *arizonae*, *houtenae*, and *indica*. Although *Salmonella* Typhimurium and *Salmonella* Enteritidis are among the most prevalent serotypes, new serovars are emerging. For instance, Lin et al. [6] identified 156 *Salmonella* isolates from chilled chicken carcasses in Taiwan, among which, *S*. Tennessee (5.1%), *S*. Schwarzengrund (20.5%), *S*. Albany (41.7%), and *S*. Kentucky (12.8%) are the frequently isolated serovars [6]. Foodborne pathogens such as *Salmonella* have developed antimicrobial resistance (AMR) over the last decade, which has led to longer hospital stays, higher treatment costs, and deaths [6].

Several variants of multidrug-resistant (MDR) *Salmonella* emerged between the late 1990s and early 2000s in humans and domestic, as well as wild, animals all over the globe [7,8,9,10,11,12,13], and their prevalence has continued to increase since then. In recent years, the emergence of MDR *Salmonella,* which is resistant to clinically relevant antibiotics such as third-generation cephalosporins and fluoroquinolones, has been increasingly recognized throughout the world [14,15].

Currently, *Salmonella*, including multidrug-resistant strains, remains a primary cause of bacterial foodborne illness, especially in low- and middle-income countries [16,17]. This is because foods are prepared with unhygienic utensils and vegetables and fruits are cultivated in contaminated environments. Moreover, foods sold in the market are often led by people who are less knowledgeable about foodborne microorganisms and the associated problems [16]. Food cross-contamination or the consumption of uncooked or raw foods such as poultry, chicken, beef, milk, vegetables, and fruits are the most common causes of human infections [18].

Several approaches to controlling *Salmonella* in foods have been found, including biological (plant extracts), physical (irradiation, autoclaved sterilization, and ozone), and chemical methods (disinfectants such as trisodium phosphate and chlorine). Nevertheless, *Salmonella* decontamination continues to pose several difficulties due to the limitations of conventional methods [19,20,21,22].

Due to their abundance, lysogenic phages represent an excellent natural resource that can be used for different biological activities. In the life cycle of these phages, their genome integrates into the host cell’s genome following infection and becomes dormant for a while, before becoming active and lysing the host cell as soon as the host environment is favorable. Currently, advanced bioinformatics tools can rapidly detect phages that harbor the lysogenic gene(s) and the factors that make them lysogenic [23,24]. The conversion of lysogenic phages to lytic phages significantly increases the diversity and efficacy of phages for therapeutic and biocontrol applications.

In 1936, Sertic and Boulgakov isolated the *Siphoviridae Salmonella* phage χ (Chi) [25]. Its primary receptor is the flagella of motile bacteria, and these kinds of phages are named flagellotropic (flagellum-dependent) phages [25]. Chi phages are distinguished from Lambda phages with their single long tail fiber, which is used to bind to the flagella on *Salmonella* cells [26,27,28]. Various studies have suggested that phage Chi and others [29,30] recognize motile flagella or pili to infect metabolically active hosts [29,30]. The genome of phage Chi is measured around 59 kb long, with 75 open reading frames (ORFs) and 56.5% GC content. There are *Salmonella* phages that are similar to Chi in their genome size, gene content, and order including FSL_SP-039, FSL_SP-124, FSLSP030, FSLSP088, and SPN19 [31,32], *Providencia stuartii* phage RedJac [33] and *Enterobacter cancerogenus* phage Enc34 [34]. There have also been identified flagellotropic non-Chi-like phages including phage CPK and CP13 which have receptors on the flagella of *Caulobacter crescentus* [29], *Bacillus subtilis* phage PBS1, and *Bacillus pumilus* phage PBP1 [35], and the recently isolated phage 7-7-1 targeting agrobacterium [36]. Flagellotropic phages may display a broad range of lytic activity (e.g., phage Chi infecting *E. coli* and *Salmonella*) [37], making them more susceptible to remediation and diagnostic tools.

In this study, we describe the isolation and characterization of two Chi-like phages from the wastewater treatment plant targeting *Salmonella enterica* serovars Typhimurium strain 85 and Enteritidis strain FORC_052. The genomic and phylogenetic features of the two newly isolated phages were closely similar to previously sequenced Chiviruses.

## 2. Materials and Methods

### 2.1. Bacterial Strains and Their Culture Conditions

The *Salmonella* serovars used in this study were kindly obtained from the Saudi Food and Drug Administration and were confirmed by 16S rRNA sequencing. The bacterial isolates, which were used as hosts and for host range analysis, were preserved at 80 °C in 50% glycerol and revived at 37 °C, overnight, in a Brain heart infusion (BHI) medium when needed.

### 2.2. Antibiotic Sensitivity Testing

The sensitivity of the host bacterium and its antibiotic resistance profile was assessed by the disk diffusion method, as described previously [38], following the CLSI guidelines [39]. The antibiotics used in this test were purchased from Oxoid^™^ Ltd. (Oxoid, Hampshire, UK) in the following concentrations: Amikacin (30 μg), Gentamicin (10 μg), Tobramycin (10 μg), Streptomycin (5 μg), Neomycin (15 μg), Ciprofloxacin (5 μg), Ampicillin (10 μg), Doxycycline (30 μg), Chloramphenicol (30 μg), Cefuroxime (30 μg), Cefotaxime (30 μg), Ceftriaxone (30 μg), Ceftazidime (30 μg) and Meropenem (10 μg). The inhibition zone diameters were measured with a caliper and the results were interpreted as resistant (R), intermediate (I), and sensitive (S) based on the CLIS standardized table.

### 2.3. Enrichment and Isolation of Phages

The raw wastewater samples were gathered from the wastewater treatment plant in Jeddah city, Kingdom of Saudi Arabia, as elucidated formerly, with slight modifications [40]. In brief, a 20 mL wastewater sample was centrifuged at 8000× *g* for 12 min at 25 °C and filtered through a 0.22-μm syringe filter (Fischer Scientific, Ottawa, ON, Canada) to remove bacteria, large particulates, and debris.

Bacteriophage enrichment, isolation, and purification were achieved as described previously [41,42]. Subsequently, 10 μL of the 0.22 μm-filtered wastewater samples were individually mixed with equal volumes of double-strength BHI broth (supplemented with 2 mM CaCl_2_) and 100 μL of mid-log cultures of either *S*. Typhimurium strain 85 or *S*. Enteritidis strain FORC_052. The enriched tubes were incubated for 48 h at 37 °C with continuous agitation at 100 rpm, and then spun down at 10,000× *g* for 10 min at 4 °C. The supernatants were collected and filtered through 0.22 μm millipore syringe filters.

The presence of bacteriophages was evaluated by spotting 10 μL of the enriched lysates onto lawns of either *S*. Typhimurium strain 85 or *S*. Enteritidis strain FORC_052, as previously described [43]. Culture dishes, spotted with phage, were examined for the presence of clear areas following overnight incubation at 37 °C. The lytic area was then cut from the top surface and immersed into 500 μL salt-magnesium (SM) buffer (0.1 M NaCl, 0.05 M Tris-HCl, and 0.01 M MgSO_4_; pH 7.5). Phage particles were allowed to diffuse out into the SM buffer through overnight incubation at room temperature with continuous shaking.

### 2.4. Phages Purification and Propagation

The purification of the isolated phages was conducted by the double agar overlay (DAO) method [44]. Single plaques showing distinct plaques morphotypes were collected from the top agar surface using sterile micropipette tips, resuspended in SM buffer, and maintained at 4 °C for 2 h. Again, the suspensions were plated using the DAO method and this procedure was repeated until morphologically identical plaques were obtained.

The full-plate lysis method was employed to propagate the purified phages [45]. The purified phages were tenfold serially diluted in SM buffer and plated using the double agar overlay method and incubated for 24–48 h at 37 °C. Plates that showed full lysis were selected and 4 mL of SM buffer was poured over the lysed area and incubated overnight at 4 °C with continuous shaking at 100 rpm. Phage suspension in the supernatant was collected by aspiration, vortexed, and centrifuged at 10,000× *g* to remove any host debris. The propagated phages were filtered through 0.22 µm and phages concentrations (PFU/mL) were evaluated using the agar overlay method.

### 2.5. Transmission Electron Microscopy (TEM)

Electron micrographs of the concentrated phage lysate (~10^12^ PFU/mL) were performed according to the method described elsewhere [46,47]. The phage lysate was dropped (~20 μL) onto 400-mesh carbon-coated grids stained with 2% (*wt*/*v*) phosphotungstic acid (pH 7.2). Thereafter, the air-dried grids were examined under the transmission electron microscope (JEM-1011, JEOL Ltd. Tokyo, Japan) at 15,000–25,000 × magnification and at an acceleration voltage of 80 kV.

### 2.6. Determination of Phages Host Range

The spectrum of killing activity of STP11 and STP13 were conducted against selected bacterial isolates by the spot assay [48]. Subsequently, 100 µL of an exponential phase culture (~10^8^ CFU mL^−1^) was mixed with 5 mL of molten BHI soft agar (0.6% agar). The preparation was then poured onto nutrient agar plates. Once dried for 5 min, 5 μL phage lysate was placed onto the top agar layer and kept at room temperature for adsorption for 20 min. The plates were then incubated overnight at 37 °C. Consequently, the plates were inspected for the presence of a growth-free area, and its presence was reflected as positive for the test, which would be further confirmed using the DAO method.

### 2.7. One-Step Growth Curve

The intracellular lytic activities of the isolated phages in a one-round replication cycle, following the procedure displayed by Bloch et al. [49], with minor modifications. The host bacterium was grown in a BHI medium at 37 °C with shaking until OD_600_ = 0.2. Afterward, 10 mL of the culture sample was spun down (4000× *g*) for 10 min at 4 °C. Following that, the pellet was resuspended in fresh LB medium. A fixed number of each bacterial host cell (5 log_10_ CFU/mL) in the mid-log-phase were inoculated with their corresponding phages at an MOI of 10. Adsorption was allowed for 5 min at 37 °C while shaking at 140 rpm, non-adsorbed virions were eliminated by rinsing 3 times with 1000 µL of BHI medium containing 3 mM sodium azide (4 °C, 4000× *g*, 10 min). Next, the pellet suspension was mixed with 25 mL of BHI medium (time 0) and incubated in a shaker incubator at 37 °C. Over the course of 60 min, approximately 100 μL aliquots were collected at intervals of 10 min. The DAO method was performed to obtain the phage titer of these aliquots after diluting them with phage buffer. Finally, the period of latency and the magnitude of virions released from one cycle (burst size) were determined. This experiment was repeated three times.

### 2.8. Thermal and pH Stability Assay

The thermal resistance of all bacteriophages was determined by heating the isolated phages at 40 °C–90 °C in a temperature-controlled water bath, and stability at 4 °C was performed in a standard refrigerator. Equal volumes of the purified phage lysate (10^8^ PFU/mL) and PBS (pH = 6.5) were incubated for 2 h. The influence of pH on the bacteriophages was assayed in nutrient broth at a pH range of between 2.0 and 14.0. The experiments were conducted at 4 °C for 2 h. The thermal and pH stabilities were determined by measuring the residual phages (PFU/mL) using the DAO technique [45].

### 2.9. Killing Assay

The lytic efficacy of the isolated phages were determined, as previously described [43]. Subsequently, overnight cultures of *S*. Typhimurium strain 85 and *S*. Enteritidis strain FORC_052 were diluted to 10^5^ CFU/mL; then, the diluted cultures were challenged individually with their corresponding phages at MOIs of 100 and 10,000. The mix was then incubated for 24 h at 37 °C. The aliquots were harvested at 0, 2, 4, 6 and 24 h post-infection (p.i.) and were serially diluted to count the surviving *Salmonella* cells.

### 2.10. Organic and Detergent Solvents

The effects of organic solvent and disinfectants on the stability of phages STP11 and STP13 were performed according to Jurczak-Kurek et al.’s protocol, with slight modifications [50]. For each solvent, 1000 µL (6 × 10^7^ PFU/mL) of phage particles were mixed with equal volumes of Sodium hypochlorite (NaOCl, 6% *v/v*) and organic solvents (70% ethanol) (Sigma Aldrich, St. Louis, MO, USA), separately, and incubated for 1 h, at 37 °C with gentle shaking. The untreated controls were prepared by mixing equal volumes of phage lysates with PBS (pH 7.4) under the same conditions. The mixtures were centrifuged at 9000 rpm for 12 min, and the phage titer was determined by the DAO method.

### 2.11. Genomic Characterization of the Isolated Phages

The genomic DNAs of phages STP11 and STP13 were extracted, purified, and quantified using the Phage DNA Isolation Kit (Biotek Corp, Norgen, ON, Canada), as per the manufacturer’s protocol. The DNA quantities were estimated using a NanoDrop ND-1000 UV-Vis spectrophotometer. The isolated DNA were stored at −20 °C for further analysis.

The purified phages’ DNA were sent to the microbial genome sequencing center (Pittsburgh, PA, USA) for sequencing. The phage genome was sequenced using a TruSeq protocol on an Illumina HiSeq platform, with 100 bp pair-end read sizes. FastQC was used to check the quality of the raw reads and the FASTQ Quality trimmer (minimum Q30 score) was used for trimming available on the public Galaxy server (https://usegalaxy.org/. Accessed on 15 August 2022). The trimmed reads were de novo assembled to a single contig with 120-fold coverage using Geneious 9.0.5 [51].

The genome map was constructed using the BLAST Ring Image Generator (BRIG) platform and the CGView online tool was used to estimate the GC content and GC skew of the genome [52]. The PHIRE platform was used to generate the promoters which are specific for the DNA sequence of the isolated phages [53].

Rho-factor independent terminator was generated by a program called ARNOLD servers [54]. GeneMarks [55] and PHAST were utilized to search the ORFs [56,57].

Protein basic local alignment search tool (Blastp) of the NCBI server (https://blast.ncbi.nlm.nih.gov/Blast.cgi. Accessed on 15 August 2022) was used to find the function of the coding sequences [58]. Putative tRNAs were predicted using GtRNAdb (http://gtrnadb.ucsc.edu. Accessed on 15 August 2022) and tRNA Scan-SE (http://lowelab.ucsc.edu/tRNAscan-SE. Accessed on 15 August 2022) [59,60] The predicted functional protein sequences were also evaluated to the food and allergy research tool (http://www.allergenonline.com. Accessed on 15 August 2022) to identify the presence of any allergic proteins. The presence or absence of virulence factors were tested by uploading all ORFs to the virulence factor database (http://www.mgc.ac.cn/VFs/. Accessed on 15 August 2022) [61,62] and the ResFinder database (http://cge.cbs.dtu.dk/services/ResFinder/. Accessed on 15 August 2022) [63].

### 2.12. Phylogenetic Analysis

The nucleotide sequence alignment and phylogenetic analysis were carried out using ClustalW and the Neighbor-joining method, employing MAFFT v.7 software [64]. The phylogenetic tree was visualized in iTOL [65].

### 2.13. Genome Comparison in a Two-Dimensional Plot

The Vector Builder’s Sequence Dot Plot tool (https://en.vectorbuilder.com/tool/sequence-dot-plot.html) was utilized to investigate close similarity genomic regions between the isolated phages sequence in comparison with the reference sequence selected from the national database (NCBI), which showed a high percent identity to the isolated phages. The two sequences were compared in a two-dimensional plot and organized on the left Y and top X axes of a two-dimensional matrix; the green and red dots represent the coordinates at which both sequences match.

### 2.14. Statistical Analysis

Statistical analysis was conducted using one-way analysis of variance (ANOVA) with the aid of GraphPad Prism software version 6 for windows (GraphPad Software Inc. San Diego, CA, USA). Statistical significance was determined at *p* < 0.05.

## 3. Results

### 3.1. Antimicrobial Sensitivity

The antibiotic resistance profiles of *S*. Typhimurium strain 85 and *S*. Enteritidis strain FORC_052 were evaluated against a selection of antibiotics (*n* = 15) belonging to eight different classes (Table 1). The data showed a resistance percentage of 46.6% and 66.6% for *S*. Typhimurium strain 85 or *S*. Enteritidis strain FORC_052, respectively, against the tested antibiotics. The tested bacteria were sensitive to the tested antibiotics belonging to the 3rd generation Cephalosporins and Carbapenems. The antibiogram data identified *S*. Typhimurium strain 85 and *S*. Enteritidis strain FORC_052 as multidrug-resistant (MDR) as they resisted many antibiotics belonging to different classes.

### 3.2. Bacteriophages Isolation and TEM Characterization

**Figure 1:**
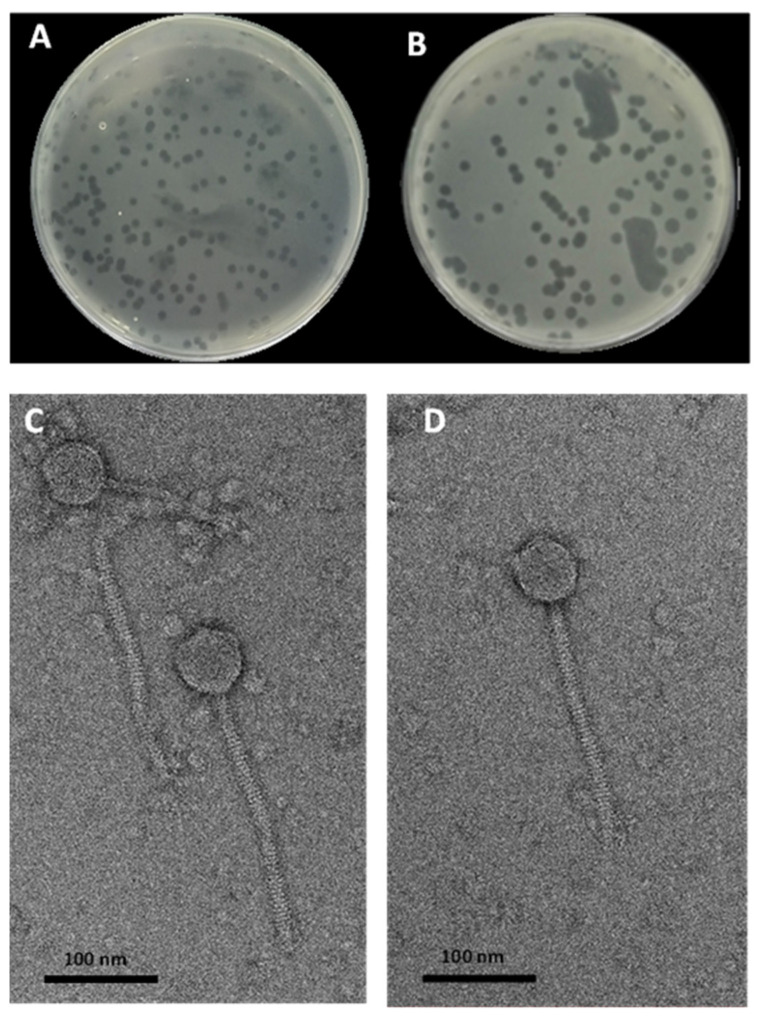
Plaque morphology and TEM of phage STP11 (**A**,**C**), and phage SEP13 (**B**,**D**). The scale bar corresponds to 100 nm.

In this study, we isolated two Chi-like *Salmonella* phages, STP11 and SEP13, respectively, against the MDR *S*. Typhimurium strain 85 and *S*. Enteritidis strain FORC_052. The presence of phages in the collected raw wastewater samples was first screened by spot assay and then further confirmed by the double agar overlying method (Figure 1A,B). A double agar overlying test was performed to determine the morphology of the plaques. STP11 and SEP13 phages produced clear, uniform-size plaques with diameters of 1.5 ± 0.5 mm and 0.5 ± 0.5 mm, respectively, on lawns of *S*. Typhimurium strain 85 and *S*. Enteritidis strain FORC_052.

The TEM observation of phages STP11 and SEP13 showed similar morphotypes, including isometric capsids with long non-contractile tails, which are features of phages belonging to the *Siphoviridiae* family under order *Caudovirales* (Figure 1C,D). Phage STP11 displayed an icosahedral head with a diameter of 65.6  ± 1.4 nm and a flexible, non-contractile tail (227 ± 1.5 nm length, 12 ± 1.5 nm width). Similarly, phage SEP13 displayed the same head and tail morphology with a capsid diameter of (64 ± 1.2) nm and a tail length of 226 ± 1.2 nm, and 11 ±  1.5 nm in width, respectively.

### 3.3. Sensitivity of the Isolated Phages to Physical and Chemical Agents

**Figure 2:**
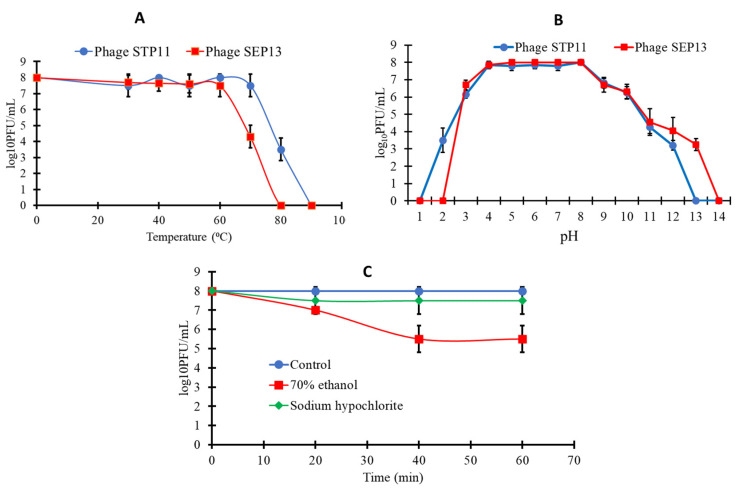
The line graph shows the stability of the isolated phage for (**A**) Thermal treatment; (**B**) pH; (**C**) Organic solvent and detergent treatment for both phages (same results obtained). Mean values ± SD for each point are displayed as log10 (PFU/mL).

The thermal stabilities of the isolated phages were assayed at neutral pH (pH 7.0). The average titers of the two phages were found to be stable (7.8 log_10_ PFU/mL) upon exposure at 4 °C, 20 °C, 40 °C, or 60 °C for 2 h. The survival rate of STP11 declined to approximately 3.7 log_10_ PFU/mL (*p* < 0.05) at 80 °C. The titer of phage SEP13 decreased to 4.5 log_10_ PFU/mL at 70 °C. We observed that almost no phages survived (*p* < 0.05) at 90 °C and 80 °C for phages STP11 and SEP13, respectively (Figure 2A).

Concerning the pH stability, as shown in Figure 2B, phages STP11 and SEP13 retained a high titer (8 log_10_ PFU/mL) from pH 4 to pH 9 for 2 h. Phage STP11 showed a significant decrease (*p* < 0.05) in titer at pH 2 and 3, with phage titers of 2.5 and 1.85 log_10_ PFU/mL, respectively, while the titer of SEP13 decreased by 1.3 log_10_ PFU/mL at pH 3. The titer of both STP11 and SEP13 became zero at strong alkaline conditions specifically at pH 13 and pH 14, respectively (*p* < 0.05). Similarly, there are no viable virions encountered at pH 1 for both phages.

The stability of the isolated phages against chemical agents was determined by subjecting it to Sodium 6% hypochlorite (NaOCl) and 70% ethanol. The results obtained from this experiment indicated that the same results were attained for both phages. According to the results, more than half of the titer of both STP11 and SEP13 was maintained at Sodium hypochlorite treatment. However, their titer was equally reduced to approximately 5.5 and 7.3 log_10_ PFU/mL in the presence of 70% ethanol and NaOCl at 60 min post-treatment, respectively, compared to the control (*p* < 0.05) (Figure 2C).

### 3.4. One-Step Growth Curve

**Figure 3:**
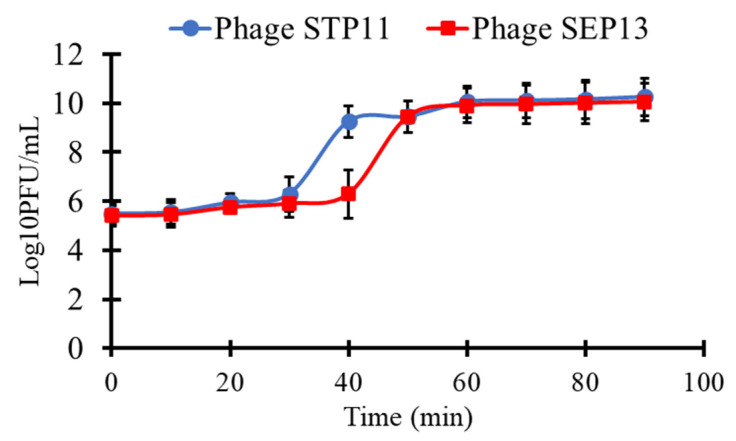
One-step growth kinetics of phages STP11 and SEP13 on their corresponding hosts. Results are displayed as means of three replicates ± SD and presented as log_10_ (PFU/mL).

One-step growth kinetics were performed to determine the latent periods and the burst sizes of the isolated phages (Figure 3). Almost all virions adsorbed onto the surface of the host cell at 30 min post-infection for both phages. The latent periods of phages STP11 and SEP13 on their isolation hosts were approximately 30 min and 40 min, respectively. Both phages completed their infection cycles within 90 min post-infection with burst sizes of 176 ± 8 and 170 ± 5 plaque-forming units per infected cell (PFU/cell), for phages STP11 and SEP13, respectively.

### 3.5. Bacterial Challenge Test

**Figure 4:**
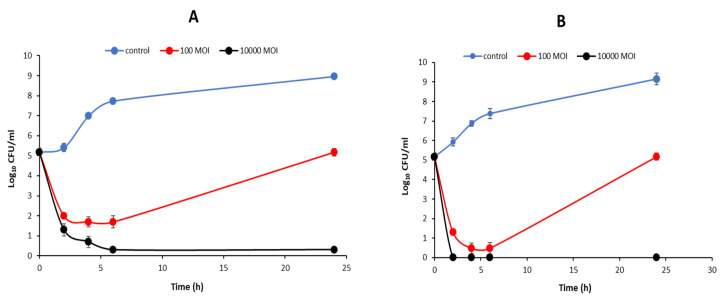
Lytic activities of phages STP11 (**A**) and Phage SEP13 (**B**) against their corresponding hosts at MOIs of 100 and 10,000. The point represents the average + SD of three replicate experiments.

The efficacy of phages STP11 and SEP13 to control the growth of *S*. Typhimurium strain 85 and *S*. Enteritidis strain FORC_052, respectively, was determined (Figure 4). In comparison to the unchallenged bacterial counts, the results showed that both phages evidently restrained the growth of their corresponding hosts, 2 h post-infection (below the detection limit, <1 log_10_ CFU/mL), when the cells were challenged with an MOI of 10,000. However, at MOI of 100, bacterial growths were inhibited for up to 6 h post-infection; then, the bacterial counts were increased gradually after 24 h p.i. This rise in survival is likely due to the rise of mutant variants early on in the growth curve, as at high MOIs all the cells become infected and the resistant ones are unaffected and quickly amplify in the nutrient-rich medium.

### 3.6. Host Range

The host range of STP11 and SEP13 was assessed by a spot test and confirmed by plaque assay. The results revealed that out of the 14 strains tested, phage STP11 was lytic against 42.8% (*n* = 6), while SEP13 showed lytic activity against 35.7% (*n* = 5) of the tested bacterial spp. The lytic activities of both phages were limited to the targeted bacterial spp. (Table 2).

### 3.7. Genomic Features

**Figure 5:**
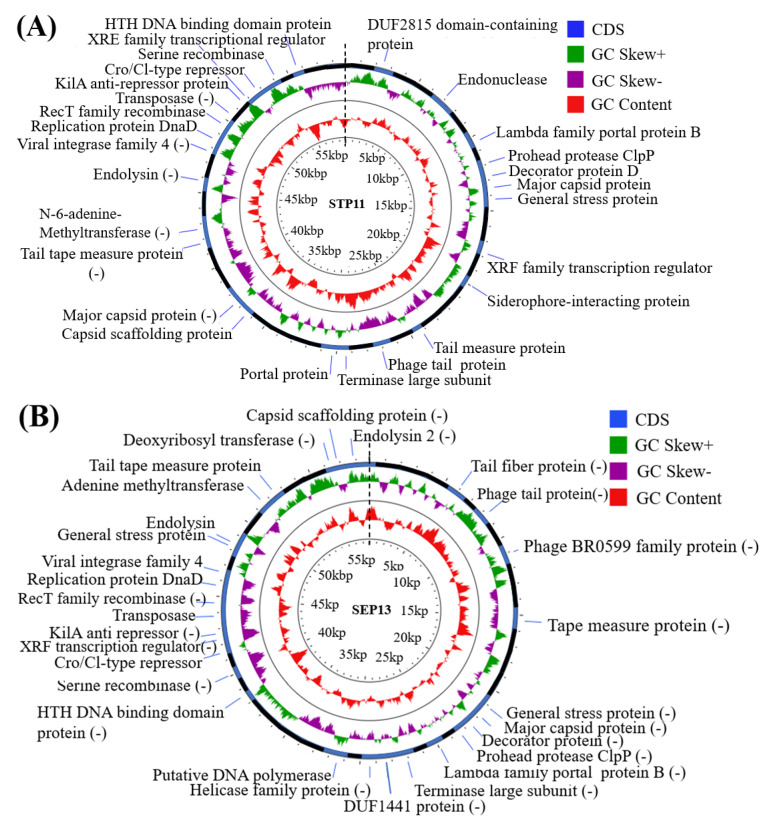
The circular genome map of phage (**A**) STP11 and (**B**) SEP13. The map was constructed and visualized using the CGView server database. The outer circle designates the ORFs of the isolated phage predicted, coupled with their putative functions. The negative sign in the parenthesis indicated the position of the strand ((main strand (not labeled), complementary strand (−)). The most inner circle marked with a red landscape designates the GC content, while the 2nd inner ring with the green and purple landscape shows the GC skew −/+ (GC-skew ((G-C)/(G+C))). The CDSs whose functions have been determined are labeled (blue color) along with their positions; however, other CDSs without labels (black color) represent hypothetical proteins. The physical map is scaled in kbp.

Phages STP11 and SEP13 have linear dsDNA genomes consisting of 58,890 bp and 58,893 bp nucleotide sequences with a G + C content of 57% and 56.5%, respectively (Figure 5). Whole genome sequences of both phages STP11 and SEP13 were deposited in the GenBank database under accession numbers OP535471 and OP535472, respectively. Bioinformatics analysis revealed that the genome of phage STP11 (Figure 5A) and SEP13 (Figure 5B) contained 70 and 71 ORFs, respectively. No gene encoding tRNA was detected in their genome. Of the 70 putative ORFs of phage STP11, 27 (38.6%) were assigned to functional genes and 43 (61.4%) were annotated as hypothetical proteins (Table 3). Similarly, 29 (40.8%) of the 71 putative ORFs of phage SEP13 were annotated as functional genes, whereas the remaining 42 (59.2%) were assigned as nonfunctional proteins. Out of the 70 putative ORFs of phage STP11, 26 (37.1%) ORFs were on the negative strand, while the other 44 (62.9%) ORFs were on the positive strand. In the genome of phage SEP13, 24 (33.8%) ORFs were situated on the positive strand, while the remaining 47 (66.2%) ORFs were found on the negative strand. In the case of the STP11 genome, most of the annotated ORFs began ATG as a starting codon, with the exception of ORFs (3, 29, 52), which began with GTG, and ORF58 which began with CTG. Detailed information regarding the annotation is provided in Supplementary Table S1. The majority of SEP13’s ORFs began with the ATG codon, with the exception of ORF68, ORF43, and ORF1, which began with GTG, CTG, and GCG, respectively (Supplementary Table S2).

The predicted functional proteins were categorized into five modules: packaging, DNA metabolism (DNA replication and encapsulation), host lysis, head, tail morphogenesis, and other proteins. The head-tail associated proteins of phage STP11 were represented by capsid scaffolding protein, major capsid protein, prohead protease ClpP, decorator protein D, putative tape measure protein, phage tail protein, and tail tape measure protein, which were encoded by ORF31, ORF10/ORF32, ORF8, ORF9, ORF19, ORF23, and ORF20/ORF44, respectively. Similar proteins were encoded by ORF69, ORF18, ORF20, ORF19, ORF11, ORF6, and ORF57/ORF11, respectively. In these functional protein categories, the putative tail fiber protein of SEP13 was encoded by ORF5. The genome of both phages STP11 and STP13 encoded three DNA replication proteins: XRE family transcriptional regulator, replication protein DnaD, and putative N-6-adenine-methyltransferase; however, in contrast to phage STP13, the genome of phage STP11 encoded three DNA replication proteins: helicase family protein, putative DNA polymerase, and Deoxyribosyl transferase, represented by ORF25, ORF27 and ORF67, respectively. Putative lambda family portal protein B and terminase large subunit were encoded by ORF7 and ORF24 for phage STP11 and ORF21 and ORF23 for phage STP13, respectively. In contrast to STP11, the genome of phage SEP13 was encoded for two cell lysis proteins: endolysin and putative endolysin 2 proteins, represented by ORF52 and ORF71, respectively. In addition to the above-mentioned functional proteins, other accessory proteins were encoded by the genome of both phages (Table 3).

No genes associated with toxin production, antibiotic resistance, or *Salmonella* virulence were identified. However, lysogenic genes such as viral integrase family 4, RecT family recombinase, transposase, kilA anti-repressor protein, putative Cro/Cl-type repressor, HTH DNA binding domain protein, and serine recombinase were identified and encoded by ORF53, ORF56, ORF58, ORF61, ORF63, ORF68, and ORF67, for STP11 and ORF48, ORF45, ORF43, ORF40, ORF38, ORF33 and ORF34 for SEP13, respectively. No rho-independent terminators were detected using ARNold (Erpin and/or RNAmotif program). No tRNA genes were predicted using the tRNAscan-SE de facto tool.

The Vector Builder’s Sequence Dot Plot tool was used to determine the degree of the close similarity between phage STP11 (Supplementary Figure S1A) and SEP13 (Supplementary Figure S1B) in comparison with the reference sequence of *Salmonella* phages ST-374 (NC_052998.1) and ER24 (NC_052999.1), selected from the national database, which showed high query coverage, accession length, and high sequence similarity (95% and 97.15%, respectively). The two-dimensional matrix confirmed that the reference and the isolated sequence showed a high sequence match (100%) in the majority of the genomic regions, as indicated by green and red dots for both reverse and forward sequences, respectively (Supplementary Figure S1).

### 3.8. Phylogenetic Analysis

**Figure 6:**
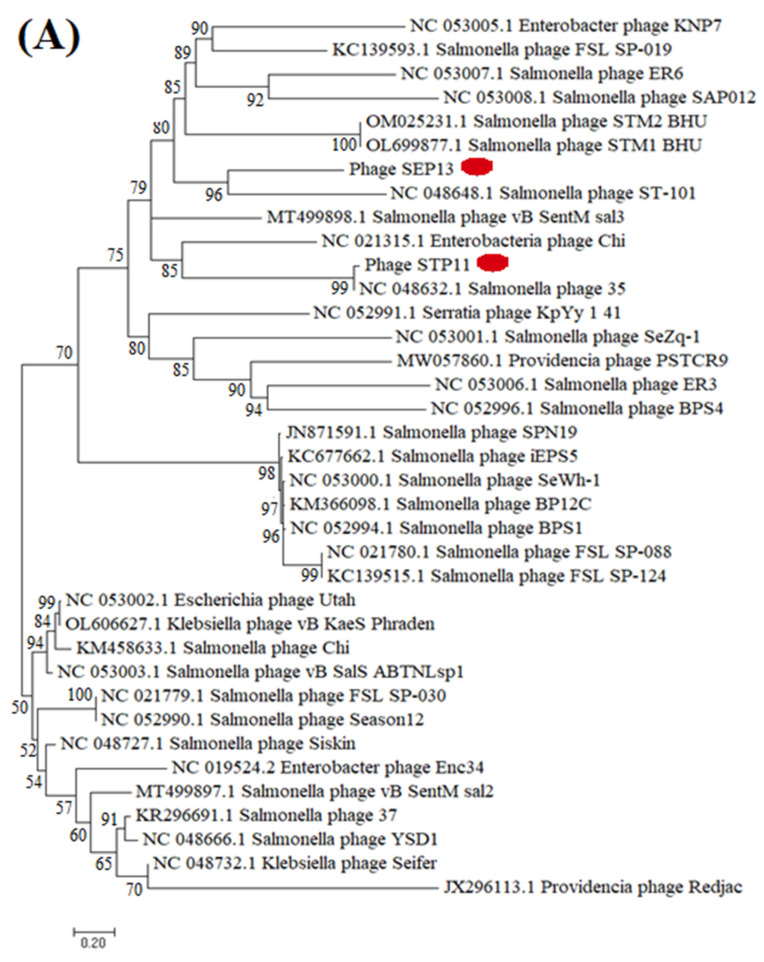

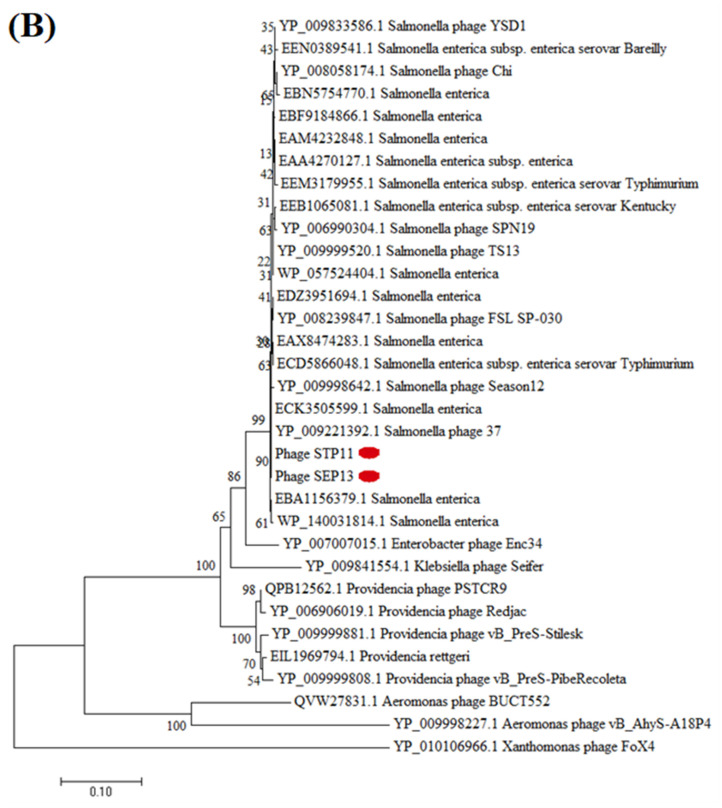
Phylogenetic tree was made using the whole genome sequence (**A**) and amino acid sequence of the major capsid protein (**B**) of phage STP11 and SEP13 and phages sharing homology sequence identity retrieved from GenBank (NCBI). The sequences were aligned using ClustalW, and the tree was built using MEGA 7 software. The evolutionary history of 33 major capsid protein amino acid sequences and 37 core genes of the whole genome sequence were aligned and inferred using the Neighbor-Joining method and 1000 bootstrap replicates. The red dot highlights the isolated phages. The scale bar represents 20% nucleotide substitution and 10% amino acid substitution percentage for the whole genomic and capsid protein map, respectively.

Phylogenetic analysis of the isolated phage was performed in comparison with the reference phages that were extracted from the national database (NCBI, BLASTp, BLASTn). The whole genome phylogenetic analysis indicated that the phage STP11 and SEP13 showed high homology with *Salmonella* phages classified in the genus Chivirus, *Siphoviridae* family under the order *Caudovirales*, which are deposited in public databases, as shown in Figure 6. Phage STP11 showed 96.89%, 96.73%, 95%, and 95% similarity with 96%, 95%, 95%, and 90% query coverage with Salmonella phage FSL SP-030 (NC_021779.1), Season12 (NC_052990.1), FSL SP-088 (NC_021780.1), and phage 35 (NC_048632.1), respectively. Similarly, SEP13 showed 98.02%, 97.83%, and 97.15% identity with similar query coverage (96%) with salmonella phage BPS1, Siskin, and ER24, respectively (Figure 6).

The phylogenetic analysis relied on the major capsid protein and indicated that the isolated phages (STP11 and SEP13) showed high sequence similarity to each other and to other Chi-like salmonella phages, whilst showing evolutionary distant from the non-salmonella Chi-like phages ((*Providenca* phage PSTCR9 (QPB12562.1)), *Providenica* phage Redjac (YP 006906019.1), Klebsiella phage Seifer (YP 009841554.1), *Aeromonas* phage vB AhyS-A18P4 (YP 009998227.1)) (Figure 6).

## 4. Discussion

*Salmonella* phages are used in many lab-oriented applications, including the creation of strains through the process called transduction [66,67], and typing them for epidemiological studies [68]. The specificity of some *Salmonella* phages and their peptides has also been used to produce strain or species-specific bio-probes for the quick detection of *Salmonella* on different food matrices [69,70] and as antibiotic alternatives to eradicate different salmonella strains on foods including chicken carcass [71,72]. In this study, we isolated novel Chi-like *Salmonella* phages from samples collected from the Jeddah wastewater treatment plant. Most Chi-like phages infect *Salmonella enterica* serovars, however, some of them are reported to be infectious for *Providencia* species [33] or *Enterobacter* species. Unfortunately, the presence of lysogenic genes in the genome of STP11 and SEP13 and related *Salmonella* Chi-like phages is the main drawback that prevents the use of these phages as therapeutic and/or biocontrol agents [73] Nevertheless, the recent advancement in phage genetic engineering allows scientists to generate strictly lytic phages using lysogenic phages [74] for diagnostic and clinical applications [75], including phage therapy in humans [76]. Phages STP11 and SEP13 belonged to the family *Siphoviridae*. Both STP11 and SEP13 phages showed comparable genome size and high sequence similarity with the *Salmonella* Chivirus. The genomic size of phage Chi is roughly 59 kb long with 75 ORFs and 56.5% GC content [31,32]. Related Chi-like phages with identical genome sizes, gene contents and orders to phage Chi include *Salmonella* phages FSL_SP-039, FSLSP030, FSLSP088, SPN19, FSL_SP-124 [31] *Providencia stuartii* phage RedJac [33] and *Enterobacter cancerogenus* phage Enc34 [34].

Successful phage therapy may depend on phage virulence, latent period, host range, burst size, obligatory lytic activities, and so on. Multiple bacterial infections are usually achieved by using broad host range phages. Some *Salmonella* bacteriophages have a wide host spectrum, but most show narrow host specificity that only infects its indicator host [54]. In this study, we found that, in comparison to phage SEP13, phage STP11 had relatively broad spectrums of antibacterial activity against the tested bacterium. With the exception of the indicator host, *S*. Typhimurium, STP11 showed the potential to infect *Salmonella enterica* subsp. arizonae, *Salmonella enterica* subsp. enterica serovar Dublin, *S*. Typhimurium (ATCC 14028), and *Salmonella enterica* subsp. enterica serovar Typhi.

According to the one-step growth cycle conducted in the present study, the isolated phages showed high burst sizes with short latent periods. According to the previous reports that the latent periods of STP11 and SEP13 were higher than the flagellotropic phage, iEPS5 (15 min) [37], but lower than the Chi-like viruses (STm101 and STm118) (>30 min) [55,77]. Phage STP11 and SEP13 had a higher burst size compared to STm101 (112 pfu/infected cell) and STm118 (48 pfu/infected cell) [77]. The use of phages with high lytic activity against large numbers of targeted bacterial populations is crucial for the large-scale biocontrol of host bacterium. This property is correlated with the large burst size. Having a large burst size for an antimicrobial agent is among the key characteristics of a good bacteriophage as burst size closely relates to phage propagation [56]. Large burst-size phages may have a selective advantage as an antibacterial agent as they can dramatically increase the initial dose several hundred-fold in a short period of time [73,78]. It is thus evident that a large burst size is a decisive advantage for their use as biocontrol agents against the tested strains.

Phage STP11 and SEP13 appeared to be stable under a broad range of temperatures (4–70 °C/80 °C) and pH values (3–12/13). These two phages did not show a significant loss of their titer for a 2 h incubation period between 4 °C to 60 °C. This finding is in agreement with the recent novel *Salmonella* Phage LPST153, which was isolated from a lake in China [79]. The two phages showed good stability at alkaline pH (pH 12), whereas reported titers of other phages were almost completely deactivated at pH 12 [80,81].

Phylogenomic analysis was conducted to investigate the relationship between our isolated phages and formerly reported Chi-like phages. In this regard, phages STP11 and SEP13 formed a monophyletic clade with each other and other Chi-like *Salmonella* phages, such as *Salmonella* phage vB SentM sal3 (MT499898.1), enterobacteria phage Chi (NC 021315.1), *Salmonella* phage 35 (NC 048632.1), and *Salmonella* phage ST-101 (NC 048648.1). The phylogenetic tree also indicated that the two candidate phages were phylogenetically distant from the non-*Salmonella* Chi-like phages. The constructed phylogenomic tree was not based on the whole genome sequence; rather, it was constructed using the 37 core genes. Hence, it may not accurately reflect their relationship. According to a report released by [61], core gene-based phylogenetic analysis represents the relationship between phages only in the high-gene flux mode. Phage-mediated horizontal gene transfer may result in genomic variation, which can obscure evolutionary relationships among phages [82,83]. Moreover, phages lack a conserved marker, universal genes, which makes it difficult to study the origin and evolutionary relationship of phages [84].

## 5. Conclusions

There have been several Chi-like *Salmonella* phages isolated so far. However, detailed molecular, as well as proteomic, studies are lacking. In this study, we have isolated and characterized two Chi-like *Salmonella* phages that were isolated using two different hosts. Based on the whole genomic sequence analysis and physicochemical parameters, the two phages shared some common characteristics which are the features of Chi-like phages. Taking into consideration the molecular analysis, the identification of specific proteins which determine the infection cycle will be crucial to broadening our understanding of these unusual phages and their interaction with the host cells. Moreover, further studies are needed to convert these phages to obligatory lytic phages by removing the lysogenic genes for better biocontrol uses.

## Figures and Tables

**Table 1:** Antibiotic sensitivity profile of S. Enteritidis strain FORC_052 and *S*. Typhimurium strain 85 against a selection of fifteen antibiotics.

Antibiotic Category	Antibiotics (Conc.)	*S*. Enteritidis Strain FORC_052 (Inhibition Zone in mm)	*S*. Typhimurium Strain 85 (Inhibition Zone in mm)
Aminoglycosides	Amikacin (30 μg)	R (10)	R (10)
	Gentamicin (10 μg)	R (9)	R (10)
	Tobramycin (10 μg)	R (9)	R (10)
	Streptomycin (5 μg)	S (20)	R (10)
	Neomycin (15 μg)	S (17)	R (12)
Fluoroquinolones	Ciprofloxacin (5 μg)	R (15)	R (10)
Penicillins	Ampicillin (10 μg)	R (10)	R (10)
Tetracyclines	Doxycycline (30 μg)	R (10)	R (10)
Phenolics	Chloramphenicol (30 μg)	R (10)	R (10)
2nd generation Cefalosporins	Cefuroxime (30 μg)	S (23)	R (10)
3rd generation Cefalosporins	Cefotaxime (30 μg)	S (20)	S (16)
	Ceftriaxone (30 μg)	S (22)	S (16)
	Ceftazidime (30 μg)	S (23)	S (18)
Carbapenems	Imipenem (10 μg)	S (25)	S (23)
	Meropenem (10 μg)	S (16)	S (15)
**Resistance percentage (%)**		**46.6%**	**66.6%**

Diameter of inhibition zones against the tested antibiotics were measured in millimeters (mm), bacterial isolates were reported as resistant (R, orange cells) or susceptible (S, green cells).

**Table 2:** Host range of STP11 and SEP13. (+) represented positive for both spot and plaque assay, (−) represented negative for both spot and plaque assay.

Category	Bacterial Species	Host Range	
		STP11	SEP13
**Enterobacteriaceae**	*Salmonella enterica* subsp. *Enterica* serovar Dublin	+	+
	*Salmonella enterica* subsp. *Enterica* serovar Enteritidis	+	+
	*Salmonella enterica* subsp. *Enterica* serovar Typhimurium	+	+
	*Salmonella enterica* subsp. arizonae	+	+
	*S*. Typhimurium (ATCC14028)	+	+
	*Salmonella enterica* subsp. *Enterica* serovar Typhi strain SRDF2	+	−
	*Escherichia coli*	−	−
	*Klebsiella pneumoniae*	−	−
	*Shigella flexneri*	−	−
	*Shigella sonnei*	−	−
	*Pseudomonas aeruginosa*	−	−
**Non- Enterobacteriaceae**	Methicillin resistant *staphylococcus aureus*	−	−
	*Streptococcus pyogenes*	−	−
	*Bacillus cereus*	−	−
**Total n (%)**	**-**	**6 (42.9%)**	**5 (35.7%)**

**Table 3:** ORFs of the isolated phages and homology to proteins databases.

Category	ORFs (Phage STP11)	ORFs (Phage SEP13)	Functions
Head-tail associated proteins	ORF31	ORF69	Capsid scaffolding protein
	ORF10, ORF32	ORF18	Major capsid protein
	ORF8	ORF20	Prohead protease ClpP
	ORF9	ORF19	Decorator protein D
	ORF19	ORF11	Putative tape measure protein
	ORF23	ORF6	Phage tail protein
	-	ORF5	Putative tail fiber protein
	ORF20, ORF44	ORF57, ORF11	Tail tape measure protein
Transcription regulator (DNA replication proteins)	ORF63, ORF17	ORF39	XRE family transcriptional regulator
	ORF56	ORF46	Replication protein DnaD
	ORF46	ORF56	Putative N-6-adenine-methyltransferase
	-	ORF25	Helicase family protein
	-	ORF27	Putative DNA polymerase
	-	ORF67	Deoxyribosyl transferase
Packaging proteins	ORF25	-	Phage portal protein
	ORF7	ORF21	Putative lambda family portal protein B
	ORF24	ORF23	Terminase large subunit
Cell lysis protein	ORF49	ORF52	Endolysin
	-	ORF71	Putative endolysin 2
Lysogenic associated protein	ORF6	-	Endonuclease
	ORF53	ORF48	Viral integrase family 4
	ORF56	ORF45	RecT family recombinase
	ORF58	ORF43	Transposase
	ORF61	ORF40	kilA anti-repressor protein
	ORF63	ORF38	Putative Cro/Cl-type repressor
	ORF67	ORF34	Serine recombinase
	ORF68	ORF33	HTH DNA binding domain protein
Other	ORF2	-	DUF2815 domain-containing protein
	-	ORF9	Phage BR0599 family protein
	-	ORF24	DUF1441 family protein
	ORF11	ORF17, ORF51	General stress protein
	ORF18	-	Siderophore-interacting protein

## Supplementary Materials

The following supporting information can be downloaded at: https://www.mdpi.com/article/10.3390/pathogens11121480/s1, Table S1: ORFs of phage STP11; Table S2: ORFs of phage SEP13; Figure S1: A two-dimensional dot plot showing the level of homology between two the phages sequences.
