# Supplementary material for: Isolation and Characterization of Chi-like Salmonella Bacteriophages Infecting Two Salmonella enterica Serovars, Typhimurium and Enteritidis
Source: Pathogens. 2022 Dec 6;11(12):1480. doi: 10.3390/pathogens11121480 (PMC9783114; doi:10.3390/pathogens11121480)
Supplement: Supplementary file 1 [file pathogens-11-01480-s001.zip › pathogens-2048133-supplementary.pdf]

pathogens

MDPI

Supplementary materials

Isolation and Characterization of Chi-like *Salmonella* Bacteriophages Infecting Two *Salmonella enterica* Serovars, Typhimurium and Enteritidis

Addisu D. Teklemariam [1], Mona G. Alharbi [1], Rashad R. Al-Hindi [1,*], Ibrahim Alotibi [2], Abdullah A. Aljaddawi [1], Sheren A. Azhari [1] and Ahmed Esmael [3,4,*]

[1] Department of Biological Sciences, Faculty of Science, King Abdulaziz University, Jeddah 21589, Saudi Arabia
[2] Health Information Technology Department, Applied College, King Abdulaziz University, Jeddah 21589, Saudi Arabia
[3] Botany and Microbiology Department, Faculty of Science, Benha University, Benha 13518, Egypt
[4] Nebraska Center for Virology, University of Nebraska-Lincoln, Lincoln, NE 68583, USA
* Correspondence: rhindi@kau.edu.sa (R.R.A.-H.); a7medesmael@gmail.com (A.E.)

Supplementary Table S1 – ORFs of phage STP11

ORFs	Strand	Start codon	Start	stop	Predicted function	E-value	Accession No
ORF1	+	ATG	774	2111	hypothetical protein	0.0	NC_021779
ORF2	+	ATG	2166	2762	DUF2815 domain-containing protein	2e-114	NC_031930
ORF3	+	GTG	2871	4868	hypothetical protein	0.0	NC_031930
ORF4	+	ATG	5204	6679	hypothetical protein	0.0	NC_031930
ORF5	+	ATG	6666	7235	Endonuclease	1e-123	NC_031930
ORF6	+	ATG	7225	9300	hypothetical protein	0.0	NC_031930
ORF7	+	ATG	9562	11241	Putative lambda family portal protein B	0.0	NC_021783
ORF8	+	ATG	11283	12554	Prohead protease ClpP	0.0	NC_021779
ORF9	+	ATG	12584	12964	Decorator protein D	8e-69	NC_021779
ORF10	+	ATG	12977	14041	Major capsid protein	0.0	NC_029045
ORF11	+	ATG	14103	14396	General stress protein	7e-49	NC_031930
ORF12	+	ATG	14399	14764	hypothetical protein	3e-62	NC_031930
ORF13	+	ATG	14764	15390	hypothetical protein	6e-112	NC_021779
ORF14	+	ATG	15387	15890	hypothetical protein	3e-92	NC_031930
ORF15	+	ATG	15904	17043	hypothetical protein	0.0	NC_029045
ORF16	+	ATG	17140	17601	hypothetical protein	3e-81	NC_031228
ORF17	+	ATG	17646	17843	XRE family transcriptional regulator	2e-32	NC_031930
ORF18	+	ATG	17836	22131	Siderophore-interacting protein	0.0	NC_031930
ORF19	+	ATG	22137	23825	hypothetical protein	0.0	NC_027991
ORF20	+	ATG	23835	24653	Tail tape measure protein	7e-162	NC_031228
ORF21	+	ATG	24665	24895	hypothetical protein	9e-39	NC_031930
ORF22	+	ATG	24895	25134	hypothetical protein	7e-41	NC_031930
ORF23	+	ATG	25124	29017	Phage tail protein	0.0	NC_021780
ORF24	+	ATG	29017	29757	Terminase large subunit	2e-144	NC_031930
ORF25	+	ATG	29767	30774	Portal protein	0.0	NC_031930
ORF26	+	ATG	30785	31747	hypothetical protein	0.0	NC_031228
ORF27	+	ATG	31761	32780	hypothetical protein	0.0	NC_031228
ORF28	+	ATG	33194	34021	hypothetical protein	2e-120	NC_031228
ORF29	+	GTG	34031	36160	hypothetical protein	0.0	NC_031930
ORF30	+	ATG	36223	36561	Capsid scaffolding protein	1e-55	NC_031930
ORF31	+	ATG	36565	37278	hypothetical protein	2e-132	NC_031930
ORF32	-	ATG	37284	37454	Major capsid protein	6e-26	NC_031930
ORF33	+	ATG	37456	37659	hypothetical protein	2e-33	NC_031228
ORF34	-	ATG	37660	38121	hypothetical protein	1e-78	NC_031228
ORF35	-	ATG	38118	38402	hypothetical protein	1e-52	NC_021783
ORF36	-	ATG	38478	39191	hypothetical protein	8e-137	NC_031930
ORF37	-	ATG	39188	39451	hypothetical protein	2e-43	NC_021779
ORF38	-	ATG	39453	39752	hypothetical protein	1e-50	NC_031930

ORF39	-	ATG	39749	40030	hypothetical protein	1e-47	NC_031930
ORF40	-	ATG	40111	40623	hypothetical protein	2e-90	NC_021780
ORF41	-	ATG	40605	*40859*	hypothetical protein	3e-46	NC_021779
ORF42	-	ATG	40849	41070	hypothetical protein	3e-36	NC_021779
ORF43	-	ATG	41067	41261	hypothetical protein	3e-32	NC_031930
ORF44	-	ATG	41272	42246	Tail tape measure protein	8e-120	NC_031930
ORF45	-	ATG	42324	43010	N-6-adenine-methyltransferase	1e-132	NC_031228
ORF46	-	ATG	43010	44110	hypothetical protein	0.0	NC_031228
ORF47	-	ATG	44107	44565	hypothetical protein	1e-81	NC_031930
ORF48	-	ATG	44709	45449	hypothetical protein	6e-146	NC_025442
ORF49	-	ATG	45645	45920	endolysin	3e-52	NC_029045
ORF50	-	ATG	45925	46158	hypothetical protein	2e-38	NC_031228
ORF51	-	ATG	46161	47234	hypothetical protein	0.0	NC_031228
ORF52	-	GTG	47215	47556	hypothetical protein	2e-57	NC_021783
ORF53	-	ATG	47543	47878	Viral integrase family 4	1e-60	NC_029045
ORF54	-	ATG	47865	48305	hypothetical protein	3e-76	NC_031228
ORF55	-	ATG	48377	48748	Replication protein DnaD	2e-63	NC_031228
ORF56	+	ATG	49509	49769	RecT family recombinase	2e-36	NC_031228
ORF57	+	ATG	49783	50322	hypothetical protein	3e-101	NC_025442
ORF58	-	CTG	50383	50502	Transposase	3e-16	NC_029045
ORF59	+	ATG	50540	51022	hypothetical protein	1e-79	NC_021779
ORF60	+	ATG	51034	51237	hypothetical protein	3e-30	NC_031228
ORF61	+	ATG	51248	51796	kilA anti-repressor protein	1e-100	NC_031228
ORF62	+	ATG	51793	52014	XRE family transcriptional regulator	5e-37	NC_031228
ORF63	+	ATG	52140	53309	Putative Cro/Cl-type repressor	0.0	NC_031228
ORF64	+	ATG	53321	53593	hypothetical protein	3e-48	NC_031228
ORF65	+	ATG	53596	53802	hypothetical protein	3e-35	NC_021779
ORF66	+	ATG	53795	54046	hypothetical protein	8e-40	NC_031228
ORF67	+	ATG	54039	54290	Serine recombinase	9e-41	NC_031228
ORF68	+	ATG	55585	55728	HTH DNA binding domain protein	2e-20	NC_025442
ORF69	-	ATG	55769	58354	hypothetical protein	0.0	NC_031930
ORF70	-	ATG	58351	58632	hypothetical protein	2e-50	NC_031228

Supplementary Table S2 – ORFs of phage SEP13

**ORF**	**Strand**	**Start codon**	**Start**	**stop**	**Predicted function**	**E-value**	**Accession No**
ORF1	-	GCG	1311	2138	hypothetical protein	2e-120	NC_031228
ORF2	-	ATG	2552	3571	hypothetical protein	0.0	NC_031228
ORF3	-	ATG	3585	4547	hypothetical protein	0.0	NC_031228
ORF4	-	ATG	4558	5499	hypothetical protein	6e-173	NC_031228
ORF5	-	ATG	5575	6315	Putative tail fiber protein	2e-144	YP_00823986
ORF6	-	ATG	6315	10208	Phage tail protein	0.0	NC_021780
ORF7	-	ATG	10198	10437	hypothetical protein	7e-41	NC_031930
ORF8	-	ATG	10437	10667	hypothetical protein	9e-39	NC_031930
ORF9	-	ATG	10679	11497	Phage BR0599 family protein	7e-162	NC_031228
ORF10	-	ATG	11507	13195	hypothetical protein	0.0	NC_027991
ORF11	-	ATG	13201	17496	Tape measure protein	0.0	YP_00999882
ORF12	-	ATG	17731	18192	hypothetical protein	3e-81	NC_031228
ORF13	-	ATG	18289	19428	hypothetical protein	0.0	NC_029045
ORF14	-	ATG	19442	19945	hypothetical protein	3e-92	NC_031930
ORF15	-	ATG	19942	20568	hypothetical protein	0.0	NC_021779
ORF16	-	ATG	20568	20933	hypothetical protein	3e-62	NC_031930
ORF17	-	ATG	20936	21229	General stress protein	7e-49	YP_00805817
ORF18	-	ATG	21291	22355	Major capsid protein	0.0	NC_029045
ORF19	-	ATG	22368	22763	Decorator protein D	8e-69	NC_021779
ORF20	-	ATG	22778	24049	Prohead protease ClpP	0.0	NC_021779
ORF21	-	ATG	24091	25770	Putative lambda family portal protein B	0.0	NC_021783
ORF22	-	ATG	25767	26021	hypothetical protein	7e-44	NC_031930
ORF23	-	ATG	26032	28107	Terminase large subunit	0.0	NC_021780
ORF24	-	ATG	28097	28666	DUF1441 family protein	7e-138	WP_01605615
ORF25	-	ATG	28653	30128	Helicase family protein	0.0	YP_01016135
ORF26	-	ATG	30175	30462	hypothetical protein	3e-52	NC_031228
ORF27	-	ATG	30464	32401	Putative DNA polymerase	0.0	YP_00999960
ORF28	-	ATG	32570	33166	hypothetical protein	9e-123	YP_00823983
ORF29	-	ATG	33221	34558	hypothetical protein	0.0	NC_021779
ORF30	-	ATG	34943	35347	hypothetical protein	0.0	NC_031930
ORF31	+	ATG	35626	35871	hypothetical protein	3e-42	NC_031930
ORF32	+	ATG	35868	38453	hypothetical protein	0.0	NC_031930
ORF33	-	ATG	38494	38637	HTH DNA binding domain protein	2e-20	NC_025442
ORF34	-	ATG	39935	40186	Serine recombinase	1e-48	YP_00922147
ORF35	-	ATG	40179	40430	hypothetical protein	8e-40	NC_031228
ORF36	-	ATG	40423	40629	hypothetical protein	3e-35	NC_021779
ORF37	-	ATG	40632	40904	hypothetical protein	3e-48	NC_031228

ORF38	-	ATG	40916	42085	Putative Cro/Cl-type repressor	0.0	NC_031228
ORF39	-	ATG	42211	42432	XRE family transcriptional regulator	5e-37	NC_031228
ORF40	-	ATG	42429	42977	kilA anti-repressor protein	1e-100	NC_031228
ORF41	-	ATG	42988	43191	hypothetical protein	3e-30	NC_031228
ORF42	-	ATG	43203	43685	hypothetical protein	1e-79	NC_021779
ORF43	+	CTG	43723	43842	Transposase	3e-16	NC_029045
ORF44	-	ATG	43903	44538	Hypothetical protein	4e-127	YP_008058147
ORF45	-	ATG	44456	44716	RecT family recombinase	2e-36	NC_031228
ORF46	+	ATG	45477	45848	Replication protein DnaD	2e-63	NC_031228
ORF47	+	ATG	45920	46360	hypothetical protein	1e-81	NC_031930
ORF48	+	ATG	46347	46682	Viral integrase family 4	1e-60	NC_029045
ORF49	+	ATG	46648	47010	hypothetical protein	3e-52	NC_021783
ORF50	+	ATG	46991	48064	hypothetical protein	0.0	NC_031228
ORF51	+	ATG	48067	48300	General stress protein	2e-38	NC_031228
ORF52	+	ATG	48305	48580	Endolysin	2e-57	NC_021783
ORF53	+	ATG	48776	49516	hypothetical protein	6e-146	NC_025442
ORF54	+	ATG	49660	50118	hypothetical protein	3e-76	NC_031930
ORF55	+	ATG	50115	51215	hypothetical protein	0.0	NC_031228
ORF56	+	ATG	51215	51901	Putative adenine-methyltransferase	7e-169	WP_14003178
ORF57	+	ATG	51979	52953	Tail tape measure protein	8e-120	NC_031930
ORF58	+	ATG	52964	53158	hypothetical protein	3e-32	NC_031930
ORF59	+	ATG	53155	53376	hypothetical protein	3e-36	NC_021779
ORF60	+	ATG	53366	53620	hypothetical protein	3e-46	NC_021779
ORF61	+	ATG	53602	54114	hypothetical protein	2e-90	NC_021780
ORF62	+	ATG	54195	54476	hypothetical protein	1e-47	NC_031930
ORF63	+	ATG	54473	54772	hypothetical protein	1e-50	NC_031930
ORF64	+	ATG	54774	55037	hypothetical protein	2e-43	NC_021779
ORF65	+	ATG	55043	55747	hypothetical protein	1e-135	NC_031930
ORF66	+	ATG	55823	56107	hypothetical protein	1e-52	NC_021783
ORF67	+	ATG	56104	56565	Deoxyribosyl transferase	1e-78	NC_031228
ORF68	-	GTG	56566	56745	hypothetical protein	4e-29	NC_031228
ORF69	-	ATG	56696	56950	Capsid scaffolding protein	3e-43	NC_031228
ORF70	-	ATG	56947	57660	hypothetical protein	2e-132	NC_031930
ORF71	-	ATG	57664	58002	Putative endolysin 2	2e-73	WP_01605612

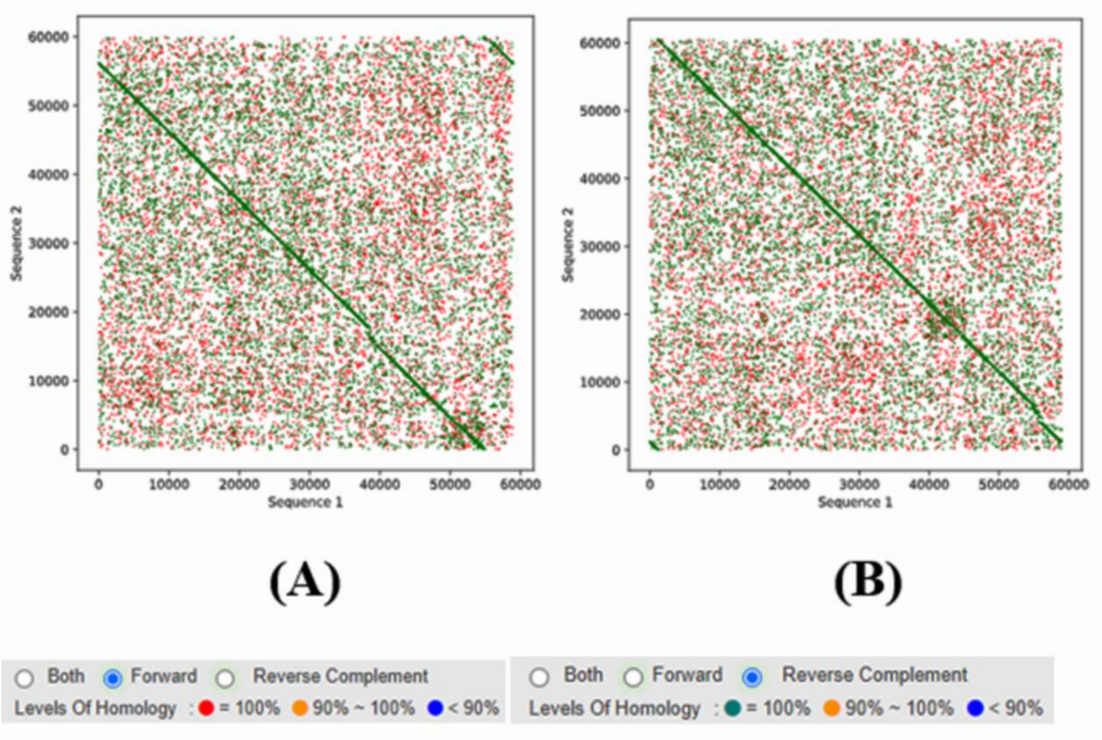

Supplementary Figure S1. A two-dimensional dot plot showing the level of homology between two the phages sequences. **(A)** sequence 1 (X -axis) represents the whole genome sequence of phage STP11 and sequence 2 (Y-axis) represents Salmonella phage ST-374 (NC_052998.1). **(B)** Sequence 1 (X-axis) represents the complete genome sequence of phage SEP13 and sequence 2 (Y-axis) represents Salmonella phage ER24 (NC_052999.1). The red and green dots represent the level of homology in both forward and reverse complement directions.
